# New Method of Parent Catheter Advancement in the Balloon Anchor Technique during Balloon-Occluded Transarterial Chemoembolization for Hepatic Tumors

**DOI:** 10.1155/2016/1957129

**Published:** 2016-05-31

**Authors:** Kei Shibuya, Hiroki Tahara, Suguru Takeuchi, Yoshinori Koyama, Yoshito Tsushima

**Affiliations:** ^1^Department of Diagnostic and Interventional Radiology, Gunma University Hospital, 3-39-22 Showa-machi, Maebashi, Gunma 371-8511, Japan; ^2^Department of Gastroenterology, Saiseikai Maebashi Hospital, 564-1 Kamishinden-machi, Maebashi, Gunma 371-0821, Japan

## Abstract

Balloon-occluded transarterial chemoembolization (B-TACE) using a microballoon catheter is a promising method for improvement of lipiodol emulsion accumulation and local control relative to conventional transarterial chemoembolization. This method has been referred to as the balloon anchor technique in previous reports. We report a new technique for successful parent catheter advancement for achievement of stable backup for the selective insertion of a microballoon catheter during B-TACE using the microballoon as an anchor, even in patients with tortuous anatomy of the hepatic and celiac arteries. Deep cannulation of parent catheters was accomplished in all three cases and complications such as vascular injury were not observed in the postprocedure angiograms.

## 1. Introduction

Balloon-occluded transarterial chemoembolization (B-TACE) using a microballoon catheter improves lipiodol emulsion accumulation in cancer nodules and, thus, local control compared with conventional transarterial chemoembolization [1–4]. Selective microballoon catheter cannulation into the feeding artery is important in reducing hepatobiliary complications related to B-TACE; however, unstable backup of the parent catheter prevents the advance of the microballoon catheter. This is caused by certain characteristics of the celiac artery. We report a new technique for successful parent catheter advancement for achievement of stable backup for the selective insertion of a microballoon catheter during B-TACE using the microballoon as an anchor, even in patients with tortuous anatomy of the hepatic and celiac artery. We call this method the balloon anchor technique. In this report, we describe our experience using the balloon anchor technique in three cases with primary hepatic malignancies.

## 2. Technique

**Figure 1:**
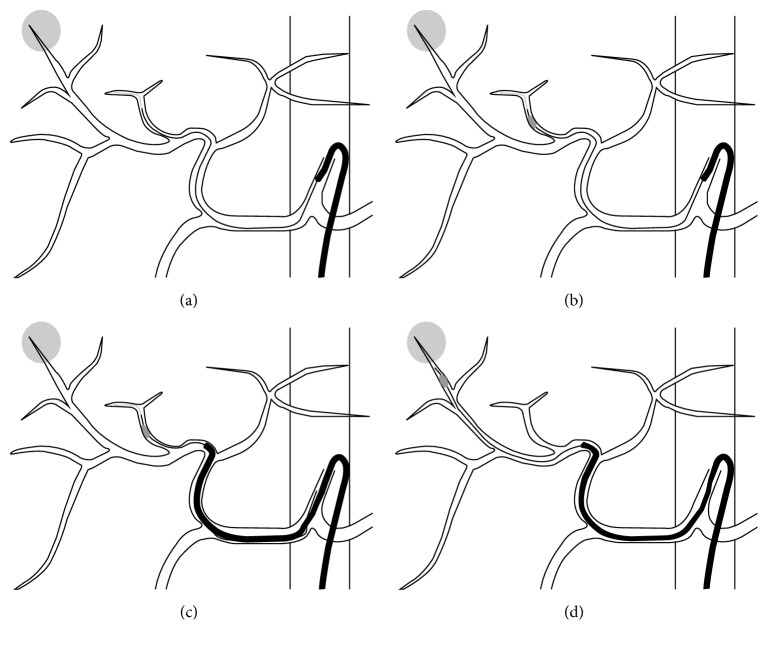
Schematic of the balloon anchor technique. (a) When selective cannulation of the feeding artery of the tumor was not possible using conventional methods, a microballoon catheter was inserted into a nontarget branch. (b) The balloon was inflated and fixed in the vessel. (c) The parent catheter was advanced along the fixed microballoon catheter. (d) The microballoon catheter was advanced into the feeding artery and selective balloon-occluded transarterial chemoembolization was performed.

The balloon anchor technique was only adapted to cases that required selective cannulation that could not be accomplished by conventional methods. The schematic of this technique is shown in Figure 1. Transfemoral access was achieved using a 5 Fr sheath, and a 5 Fr catheter was used as the parent catheter for the microballoon catheter. An over-the-wire microballoon was inserted and inflated in a peripheral branch of the hepatic artery that was not a feeding artery for the target tumors. Following inflation and fixation of the microballoon catheter, the parent catheter was advanced to the proper or common hepatic artery by gentle retraction of the microballoon catheter as a guide wire. Based on stable backup of the parent catheter, selective B-TACE was then achieved in all cases. Postprocedure diagnostic angiography was performed to confirm the absence of vascular complications related to this technique. Written informed consent was obtained from all individual participants before the procedure. For this type of study, formal consent is not required.

## 3. Case Presentation

### 3.1. Case  1

**Figure 2:**
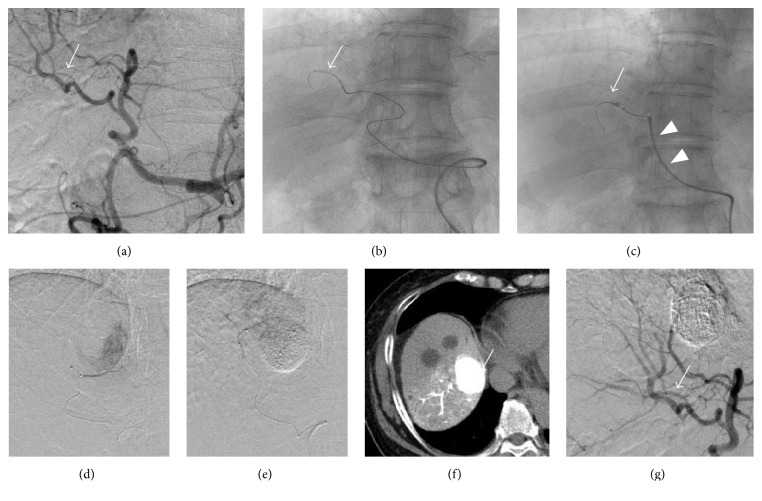
((a) and (b)) A 1.8 Fr tip coaxial microballoon catheter was inserted into a branch in segment five (arrow) that was unrelated to the feeding arteries of the tumor in segment eight. (c) The microballoon was inflated in the vessel (arrow) and the parent catheter was advanced into the right hepatic artery (arrowheads). ((d), (e), and (f)) Selective transarterial chemoembolization using miriplatin was performed and dense lipiodol deposition in the tumor was visible in the postprocedure computed tomography images. (g) Including the vessel in which the microballoon was inflated (arrow), no vascular complications were confirmed in the postprocedure angiogram.

A 70-year-old woman with liver cirrhosis caused by the hepatitis C virus was admitted for the treatment of a hepatocellular carcinoma (HCC). Although the liver tumor was solitary, surgical resection was avoided because of poor liver function and transarterial chemoembolization (TACE) was planned as the initial treatment. Diagnostic angiography was performed using a 5 Fr shepherd-hook catheter (FANSAC: Terumo Clinical Supply, Gifu, Japan). Digital subtraction angiography, computed tomography (CT) during arterial portography and CT hepatic arteriography demonstrated a hypervascular tumor in segment eight. Because the size and location of the tumor was not suitable for local ablation therapy, B-TACE was planned for better local control than could be achieved using conventional TACE. We exchanged the 5 Fr shepherd-hook catheter for a 5 Fr Cobra-shaped catheter (Selecon SNCC: Terumo Clinical Supply) for deep cannulation into the hepatic artery. Although we tried to advance the catheter to the proper hepatic artery, it would not cross because of the vessel's tortuous anatomy. A 1.8 Fr tip coaxial microballoon catheter (Logos: PIOLAX, Kanagawa, Japan) was inserted into the right hepatic artery via the parent catheter. However, selective cannulation into the tumor feeders was not possible because of the tortuous arterial anatomy and unstable backup of the parent catheter. Deep cannulation and stable backup of the parent catheter were essential in this situation, but the parent catheter could not be advanced using a 0.035-inch hydrophilic coated guide wire (Radifocus, Terumo, Tokyo, Japan). Therefore, we selected the balloon anchor technique for the advancement of the parent catheter (Figure 2). A microballoon catheter with a 0.014-inch guide wire (Transend EX: Boston Scientific, Marlborough, MA, USA) was inserted into an intrahepatic branch that was unrelated to the feeding artery, via the parent catheter. The microballoon was then inflated and fixed in the vessel. Using the microballoon catheter as a guide wire, we advanced the parent catheter into the right hepatic artery through the tortuous proper hepatic artery. Finally, we performed super-selective B-TACE into the feeding arteries of the tumor with stable support of the parent catheter.

### 3.2. Case  2

**Figure 3:**
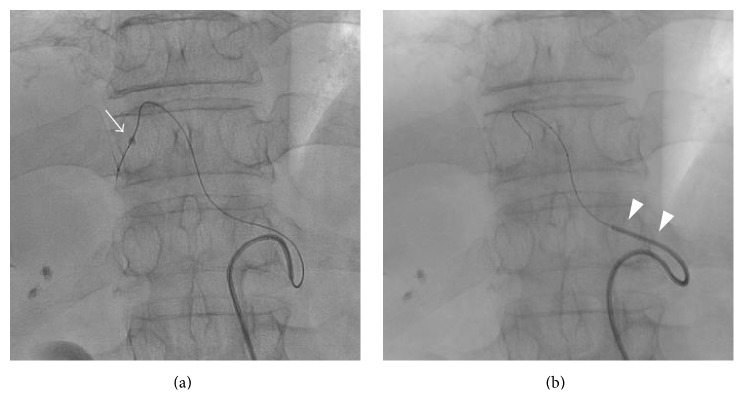
(a) A microballoon catheter was inserted into a branch of the left medial segment and inflated (arrow). (b) The 5 Fr catheter was advanced into the common hepatic artery (arrowheads). In this figure, the microballoon had already been deflated.

The second case was a 60-year-old man with hypovascular hepatic tumors diagnosed as poorly differentiated adenocarcinoma of unknown origin. Surgical resection was not indicated and cisplatin-based transarterial infusion chemotherapy, followed by TACE under feeder occlusion using a microballoon catheter, was planned for the treatment of the main tumor in the right liver lobe. Diagnostic angiography performed using a 5 Fr modified spiral-shaped catheter (Gridecath, Terumo, Tokyo, Japan) revealed stenosis at the origin of the celiac artery, and collateral blood flow from the superior mesenteric artery to the proper hepatic artery via dilated arterial arcades around the pancreas head. Deep cannulation of the 5 Fr catheter using a 0.035-inch guide wire was impossible; consequently, we selected the balloon anchor technique. As in Case 1, a 1.8 Fr tip microballoon catheter was inserted into the left hepatic artery via the parent catheter and the 5 Fr catheter was then successfully advanced into the common hepatic artery (Figure 3). Subsegmental B-TACE was then performed and good drug distribution was achieved.

### 3.3. Case  3

An 80-year-old woman presented with a large recurrent HCC in the medial segment after conventional TACE. We planned B-TACE for the recurrent tumor to achieve superior drug distribution to that of the previously used TACE technique. Celiac angiography was carried out using a 5 Fr Cobra-shaped catheter. However, as a result of severe arteriosclerosis, backup of the 5 Fr catheter was unstable and deep cannulation by means of guide wires was impossible. Similar to Cases 1 and 2, a 1.8 Fr tip microballoon catheter was inserted into a branch in segment six and fixed by inflation of the balloon. The 5 Fr catheter was then successfully advanced into the proper hepatic artery using the balloon anchor technique and the recurrent HCC was treated by means of selective B-TACE. Obvious vascular injury was not observed in the postprocedure angiogram in all three cases.

## 4. Discussion

In the present case report, we describe the balloon anchor technique, a new technique that assists the advancement of parent catheters through regions of tissue with difficult anatomy, to achieve reliable support of catheters during B-TACE for hepatic tumors. Deep cannulation of the parent catheter facilitated clear angiograms as a result of high-flow injection of contrast medium, and selective TACE was supported by stable backup. Techniques to stabilize the guiding catheter using a balloon as the anchor or guide wire have been reported previously in the fields of coronary intervention, neurointervention, and endoscopic intervention [5–8]. Our new technique was developed based on the results reported in these previous studies.

Reduction of mean arterial stump pressure by means of balloon occlusion is important in obtaining dense lipiodol accumulation during B-TACE [2]; consequently, selective insertion of a microballoon is required to prevent inflow from the collateral arteries. However, the flexibility and selectivity of microballoon catheters are lower than conventional microcatheters; in addition, it is sometimes difficult to advance a microballoon catheter because of kick-back movement in the parent catheter. All three of our patients had difficult anatomy regarding reliable support of the parent catheters during B-TACE. Also, 3 Fr or thinner microballoon catheters are required for the performance of B-TACE using a standard coaxial system. As a result of this structural limitation, angiograms from the proper or left/right hepatic artery via a microballoon catheter are insufficient in most cases; this is because of the lower injection rate of the contrast medium. Although these problems can usually be resolved by advancing the parent catheter into the hepatic artery, this is occasionally impossible because of tortuous anatomy or angulation of the celiac artery. The balloon anchor technique is useful in these situations. We consider that the balloon anchor technique could also be used to improve catheter backup in embolization for aneurysms or other abdominal vascular interventions, and further investigation is proceeding.

Additional study will be required to confirm the safety of the balloon anchor technique; however, technique-related complications such as vascular injuries were not observed in the postprocedure angiograms in our three cases. In addition, we inserted the anchor balloon into a nontarget artery and inflated it gently to avoid vascular injury to the feeding artery.

In conclusion, the balloon anchor technique can be applied to TACE for hepatic malignancies. It is a promising choice when selective catheter insertion is not readily achievable by other methods and more stable backup is required.
